# Evaluation of the Revogene Carba C Assay for Detection and Differentiation of Carbapenemase-Producing Gram-Negative Bacteria

**DOI:** 10.1128/JCM.01927-19

**Published:** 2020-03-25

**Authors:** Delphine Girlich, Marine Laguide, Laurent Dortet, Thierry Naas

**Affiliations:** a EA7361 “Structure, dynamic, function and expression of broad spectrum β-lactamases,” Université Paris-Saclay, LabEx Lermit, Faculty of Medicine, Le Kremlin-Bicêtre, France; b Evolution and Ecology of Resistance to Antibiotics Unit, Institut Pasteur-APHP, Paris, France; c Bacteriology-Hygiene Unit, Assistance Publique-Hôpitaux de Paris, Bicêtre Hospital, Le Kremlin-Bicêtre, France; d Associated French National Reference Center for Antibiotic Resistance: Carbapenemase-producing Enterobacteriaceae, Le Kremlin-Bicêtre, France; University of Iowa College of Medicine

**Keywords:** multiplex PCR, Gram-negative bacteria, real time, screening

## Abstract

The Revogene Carba C assay (formerly GenePOC Carba assay) is a multiplex nucleic acid-based *in vitro* diagnostic test intended for the detection of carbapenemase-producing *Enterobacterales* (CPE) from cultured colonies. This assay was evaluated directly on colonies of 118 well-characterized *Enterobacterales* with reduced susceptibility to carbapenems and on 49 multidrug-resistant (MDR) Pseudomonas aeruginosa and 40 MDR Acinetobacter baumannii isolates. The Revogene Carba C assay’s performance was high, as it was able to detect the five major carbapenemases (NDM, VIM, IMP, KPC, and OXA-48). In *Enterobacterales*, sensitivity and specificity were 100%. When extrapolating the results to the French CPE epidemiology between 2012 and 2018, this assay would have detected 99.28% of the 9,624 CPE isolates sent to the French NRC, missing 69 CPE isolates (2 GES-5, 10 OXA-23, 2 TMB-1, 1 SME-4, 53 IMI, and 1 FRI). The overall sensitivity and specificity for CP P. aeruginosa were 93.7 and 100%, respectively, as two rare IMP variants (IMP-31 and -46) were not detected. Extrapolating these results to the French epidemiology of CP P. aeruginosa in 2017, 93.3% would have been identified, missing only 1 DIM and 10 GES variants. The Revogene Carba C assay accurately identified the targeted carbapenemase genes in A. baumannii, but when extrapolating these results to the French CP A. baumannii epidemiology of 2017, only 12.50% of them could be detected, as OXA-23 is the most prevalent carbapenemase in CP A. baumannii. The Revogene Carba C assay showed excellent sensitivity and specificity for the five most common carbapenemases regardless of the bacterial host. It is well adapted to the CPE and CP P. aeruginosa epidemiology of many countries worldwide, which makes it suitable for use in the routine microbiology laboratory, with a time to result of ca. 85 min for eight isolates simultaneously.

## Abstract (precis)

The Revogene Carba C assay (formerly GenePOC Carba assay) is a multiplex nucleic acid-based *in vitro* diagnostic test intended for the detection of carbapenemase-producing *Enterobacterales* (CPE) from cultured colonies. This assay was evaluated directly on colonies of 118 well-characterized *Enterobacterales* with reduced susceptibility to carbapenems and on 49 multidrug-resistant (MDR) Pseudomonas aeruginosa and 40 MDR Acinetobacter baumannii isolates.

## INTRODUCTION

Carbapenemase-producing (CP) Gram-negative bacteria (CP-GNB) (*Enterobacterales* [CPE], Pseudomonas aeruginosa [CP-Pa], and Acinetobacter baumannii [CP-Ab]) have increasingly been reported worldwide (1, 2). Carbapenem resistance may be the result of combined mechanisms of both outer membrane permeability defects (e.g., porin defects) and noncarbapenemase β-lactamase (e.g., acquired or overexpressed chromosome-encoded cephalosporinase and/or extended-spectrum β-lactamase) and carbapenemase production (1). The most clinically relevant carbapenemases belong to either Ambler class A or B (metallo-β-lactamases [MBLs]) or Ambler class D (carbapenem-hydrolyzing class D β-lactamases [CHDLs] or oxacillinases) (3). The Ambler class A carbapenemases include the worldwide-disseminated Klebsiella pneumoniae carbapenemase (KPC)-type enzymes and, to a lesser extent, GES, SME, IMI, NMC-A, and FRI-1 variants (4). MBLs are divided into 3 main groups, the IMP, VIM, and NDM types (3, 5). CHDLs mostly belong to (i) the OXA-48 group in *Enterobacterales* (including a growing number of variants) (1), (ii) OXA-23, OXA-24/-40, OXA-58, OXA-143, and the overexpressed intrinsic OXA-51-like enzymes in A. baumannii, and (iii) OXA-198 in P. aeruginosa (6–9).

As CP organisms (CPOs) are a major health issue, rapid confirmation of carbapenemase production is essential not only for effective therapy but also for the prompt implementation of infection control measures able to prevent their dissemination (10). Screening protocols are based mainly on cultures of rectal swab specimens on selective media (11, 12), followed by phenotypic tests to confirm a carbapenem-hydrolyzing activity, such as the Carba NP test (13), the rapid carbapenem inactivation method (rCIM) (14), lateral flow immunoassays (15, 16), imipenem hydrolysis detected by matrix-assisted laser desorption ionization–time of flight (MALDI-TOF) (17), the BYG test (18), and the β-Carba test (19) or disk diffusion synergy test (DDST) for detection of MBLs and KPCs (e.g., meropenem disks alone and meropenem disks supplemented with aminophenylboronic acid, dipicolinic acid, or cloxacillin) (20). High-level temocillin resistance is a useful phenotypic trait for OXA-48 detection (21). Culture of rectal swab specimens followed by confirmation testing is a long process and often is not compatible with rapid implementation of reinforced hygiene measures (22).

Molecularly based techniques such as PCR and whole-genome sequencing remain the gold standard for the precise identification of carbapenemase genes (22, 23). Molecular methods are now available for detecting carbapenemase genes from bacterial cultures but also directly from clinical specimens in less than an hour (22). Several assays are commercially available. Some require DNA extraction from rectal swab specimens prior to amplification, such as the PCR-based Amplidiag CARBA+MCR assay (Mobidiag, Paris, France) (24), the Amplidiag CARBA+VRE assay (Mobidiag, Paris, France) (25), or the Check-Direct CPE assay (Check-Points, Wageningen, The Netherlands) (26), while others do not require DNA extraction, such as the Eazyyplex SuperBug CRE (Amplex Biosystems GmbH, Gießen, Germany) (27). Others are fully automated, such as CRE ELITe MGB kits on the InGenius platform (Elitech, Les Ulis, France) (28), the BD MAX Checkpoint CPO assay (29), and the GeneXpert Carba-R assay (Cepheid, Sunnyvale, CA, USA) (30, 31). Most of these assays target the “big 5” carbapenemases, i.e., KPC, OXA-48-like, NDM, VIM, and IMP, which represent more than 99% of the carbapenemases produced by *Enterobacterales* in countries such as France (32). Very recently, the biological performance of the Revogene Carba C assay (Meridian Bioscience, Cincinnati, OH, USA) (formerly GenePOC Carba assay [GenePOC, Québec, Canada]) has been compared to that of the Xpert Carba-R assay for the detection of the *bla*_KPC_, *bla*_NDM_, *bla*_VIM_, *bla*_OXA-48-like_, and *bla*_IMP_ genes from pure colonies of *Enterobacterales* (33). The four most common carbapenemases (NDM, KPC, OXA-48-like, and VIM) were detected with 100% sensitivity by both tests, but for IMPs, the Revogene Carba C assay showed 100% sensitivity, while that of the Xpert Carba-R assay was only 44.4% (33).

In the present study, we have evaluated the performance of the Revogene Carba C assay for the qualitative detection of the most widespread carbapenemase genes (*bla*_KPC_, *bla*_NDM_, *bla*_VIM_, *bla*_IMP_, and *bla*_OXA-48_) on bacterial colonies of CPE but also on nonfermenting CPOs (P. aeruginosa and A. baumannii).

## MATERIALS AND METHODS

### Bacterial isolates.

Bacterial isolates were from the Bicêtre strain collection and correspond to resistance mechanisms identified in France during the last 10 years. Among the 118 cultured colonies of *Enterobacterales* tested, 92 produced carbapenemases targeted by the assay (KPC [*n* = 17], VIM [*n* = 9], IMP [*n* = 10], NDM [*n* = 13], OXA-48 [*n* = 30], and multiple carbapenemases [*n* = 13]), 13 were carbapenem-resistant *Enterobacterales* but not CPE, 13 were nontargeted CPE, and two produced OXA-48-like carbapenemases (OXA-405 and OXA-163) that lack carbapenem-hydrolytic activity and therefore are not carbapenemases (34, 35) (Table 1). Among the 49 cultured colonies of *Pseudomonas* sp., 31 produced targeted carbapenemases (4 KPC, 8 VIM, 17 IMP, and 2 NDM), 11 were carbapenem-resistant *Pseudomonas* sp. but not CP-Pa, and 7 were nontargeted CP-Pa (Table 2). Among the 40 cultured colonies of Acinetobacter sp., 17 produced targeted carbapenemases (1 VIM, 4 IMP, and 12 NDM), 10 were non-carbapenemase producers, and 13 were nontargeted CP-Ab (Table 3). Bacteria were plated on either MH or CarbaSmart agar (bioMérieux, Marcy-l’Etoile, France) or on URI4 (Bio-Rad, Marne la Coquette, France).

**TABLE 1:** Global performances of the Revogene Carba C assay on colonies of *Enterobacterales* grown on MH agar (*n* = 118)

Category and carbapenemase and/or species (no. of isolates)	β-Lactamase content (no.)^*a*^	Revogene Carba C assay results^*b*^				
		KPC	OXA-48	NDM	VIM	IMP
Carbapenem-resistant non-carbapenemase producers (13)						
Escherichia coli (4), Enterobacter cloacae (2), Citrobacter freundii (1), K. pneumoniae (5), Enterobacter aerogenes	Case (4), Pase (1), CTX-M-2 (1), CTX-M-14 (1), CTX-M-15 (4), TEM-24 (1), SHV-38 (1)	−	−	−	−	−
Nontargeted-carbapenemase producers (13)						
Serratia marcescens (2), E. cloacae (5), Enterobacter asburiae (1), C. freundii (2), K. pneumoniae (1), Proteus mirabilis (2)	Sme-2 (1), Sme-4 (1), IMI-1 (1), IMI-2 (1), FRI-1 (1), GES-5 (1), GES-6 (1), OXA-23 (1), OXA-58 (1), OXA-372 (1), LMB-1 (1), TMB-1 (1), GIM-1 (1)	−	−	−	−	−
Targeted-carbapenemase producers (92)						
KPC type (17)						
E. coli (9), K. pneumoniae (4), E. cloacae (2), C. freundii (1), S. marcescens (1)	KPC-2 (7), KPC-3 (4), KPC-5 (1), KPC-6 (1), KPC-7 (1), KPC-14 (1), KPC-28 (1), KPC-39 (1)	+	−	−	−	−
NDM type (13)						
E. coli (9), K. pneumoniae (2), Providencia rettgeri (1), Salmonella enterica (1)	NDM-1 (6), NDM-4 (1), NDM-5 (1), NDM-6 (1), NDM-7 (1), NDM-9 (1), NDM-11 (1), NDM-19 (1)	−	−	+	−	−
VIM type (9)						
E. coli (2), K. pneumoniae (3), E. cloacae (2), C. freundii (2)	VIM-1 (4), VIM-2 (2), VIM-4 (2), VIM-19 (1)	−	−	−	+	−
IMP type (10)						
E. coli (3), K. pneumoniae (3), E. cloacae (1), S. marcescens (2), C. freundii (1)	IMP-1 (2), IMP-8 (4), IMP-10 (1), IMP-11 (1), IMP-14 (1), IMP-58 (1)	−	−	−	−	+
OXA-48-like with carbapenemase activity (28)						
E. coli (13), K. pneumoniae (12), E. cloacae (1), Citrobacter koseri (1), C. freundii (1)	OXA-48 (8), OXA-162 (1), OXA-181 (4), OXA-204 (5), OXA-232 (2), OXA-244 (4), OXA-484 (1), OXA-517 (1), OXA-519 (1), OXA-793 (1)	−	+	−	−	−
OXA-48-like without carbapenemase activity (2)						
K. pneumoniae (1), S. marcescens (1)	OXA-163 (1), OXA-405 (1)	−	−	−	−	−
Multiple (13)						
E. coli (2), K. pneumoniae (2),	NDM-1 + OXA-48 (1), NDM-1 + OXA-232 (1), NDM-5 + OXA-232 (1), NDM-5 + OXA-181 (1)	−	+	+	−	−
E. coli (1)	NDM-1 + VIM-2 (1)	−	−	+	+	−
K. pneumoniae (1)	NDM-5 + OXA-181 + VIM-1 (1)	−	+	+	+	
C. freundii (2), E. cloacae (1)	VIM-4 + OXA-48, VIM-1 + OXA-505	−	+	−	+	−
E. cloacae (2), K. pneumoniae (1)	NDM-7 + KPC-4, NDM-4 + KPC-2	+	−	+	−	−
E. coli (1)	OXA-48 + KPC-28 (1)	+	+	−	−	−

a Case, overexpressed cephalosporinase; Pase, overexpressed penicillinase.

b −, no amplification; +, amplification. Gray, matching results.

**TABLE 2:** Global performance of the Revogene Carba C assay on *Pseudomonas* sp. strains (*n* = 49)

Category and carbapenemase and/or species (no. of isolates)	β-Lactamase content (no.)	Revogene Carba C assay results^*b*^				
		KPC	OXA-48	NDM	VIM	IMP
Carbapenem-resistant non-carbapenemase producers (11)						
P. aeruginosa (2)	Mex A/B-OprM (1), Mex C/D-OprJ (1)	*_−_*	−	−	−	−
P. aeruginosa (4)	Case^*a*^ + OprD deficient	−	−	−	−	−
P. aeruginosa (5)	GES-1 (1), VEB-1 (1), PER-1 (1), OXA-32 (1), PME-1 (1)	−	−	−	−	−
Nontargeted-carbapenemase producers (7)						
P. aeruginosa (7)	GIM-1 (1), AIM-1 (1), SPM-1 (1), DIM-1 (1), GES-2 (1), GES-5 (1), OXA-198 (1)	−	−	−	−	−
Targeted-carbapenemase producers (31)						
KPC type						
P. aeruginosa (4)	KPC-2 (4)	+	−	−	−	−
NDM type						
P. aeruginosa (2)	NDM-1 (2)	−	−	+	−	−
VIM type						
P. aeruginosa (5), P. putida (1), P. stutzeri (1), P. fluorescens (1)	VIM-1 (2), VIM-2 (6), VIM-4 (1)	−	−	−	+	−
IMP type						
P. aeruginosa (13), P. putida (1), P. stutzeri (1)	IMP-1 (4), IMP-2 (1), IMP-13 (2), IMP-15 (1), IMP-19 (1), IMP-26 (1), IMP-29 (1), IMP-39 (1), IMP-56 (1), IMP-63 (1), IMP-71 (1)	−	−	−	−	+
P. aeruginosa (1), P. putida (1)	IMP-31 (1), IMP-46 (1)	−	−	−	−	−

a Case, overexpressed cephalosporinase.

b −, no amplification; +, amplification. Dark gray, discrepant result; light gray, matching results.

**TABLE 3:** Global performances of the Revogene Carba C assay on Acinetobacter sp. strains (*n* = 40)

Category and carbapenemase and/or species (no. of isolates)	β-Lactamase content (no.)	Revogene Carba C assay results^*a*^				
		KPC	OXA-48	NDM	VIM	IMP
Non-carbapenemase producers (10)						
A. baumannii (10)	WT (2), GES-11 (1), GES-12 (1), RTG-4 (1), SCO-1 (1), VEB-1 (2), PER-1 (1), OXA-21 (1)	−	−	−	−	−
Nontargeted-carbapenemase producers (13)						
A. baumannii (13)	SIM-1 (1), OXA-143 (1), OXA-243 (1), GES-14 (1), OXA-51-like (+ IS*Aba*1) (2)^*b*^, OXA-23 (3), OXA-40 (2), OXA-58 (2)	−	−	−	−	−
Targeted-carbapenemase producers						
NDM type						
A. baumannii (7)	NDM-1 (5), NDM-2 (1), NDM-9 (1)	−	−	+	−	−
IMP type						
A. baumannii (1), A. nosocomialis (1), A. junii (1)	IMP-1 (2), IMP-14 (1)	−	−	−	−	+
Multiple						
A. baumannii (4)	NDM-1 + OXA-23 (4)	−	−	+	−	−
A. baumannii (1)	NDM-1 + ISAba1OXA-51 (1)	−	−	+	−	−
Acinetobacter sp. genomospecies 16 (1)	VIM-4 + OXA-23 (1)	−	−	−	+	−
A. baumannii (1)	IMP-4 + OXA-58 (1)	−	−	−	−	+

a −, no amplification; +, amplification. Gray, matching results.

b OXA-51-like chromosomally encoded class D β-lactamases have a carbapenemase activity when overexpressed, i.e., when IS*Aba*1 is located upstream from the *bla*_OXA-51-like_ genes in A. baumannii.

### Revogene Carba C assay.

The Revogene Carba C assay was used as recommended by the manufacturer on fresh overnight bacterial colonies. Bacteria to be tested were resuspended in physiological water until a 0.5 McFarland suspension was reached. Fifteen microliters of the bacterial suspension was added to the sample buffer tube. After subsequent mixing by vortexing for 15 s, 200 μl of sample buffer containing the bacteria was transferred into the sample loading chamber of the microfluidic cartridge. As recommended in the manufacturer’s instructions, 8 cartridges were used for every run in the Revogene instrument, using blank cartridges when fewer than 8 samples were processed. Extraction, real-time PCR, and detection are integrated, providing results in 70 min for 8 isolates simultaneously; an additional 15 min was required for sample preparation.

### Statistical analysis.

The sensitivity and specificity of the Revogene Carba C assay were calculated with their respective 95%CIs using the free software vassarStats website for statistical computation (http://vassarstats.net/). The gold standard was PCR followed by sequencing. The Youden index (sensitivity [%] + specificity [%] − 100) calculates the efficiency of a diagnostic test; a Youden score of 100 indicates a perfect test.

## RESULTS AND DISCUSSION

The Revogene Carba C assay was tested on a collection of 118 well-characterized *Enterobacterales* with reduced susceptibility to carbapenems and on 49 P. aeruginosa and 40 Acinetobacter sp. isolates resistant to carbapenems and expressing various β-lactamases (Tables 1 to 3).

During this evaluation, the Revogene Carba C assay was used as recommended by the manufacturer, on fresh overnight bacterial colonies. The procedure for sample preparation was easy to perform, taking ca. 15 min per eight samples, with a run time of approximately 70 min for one to eight samples simultaneously on the Revogene instrument. The results were interpreted by the Revogene software and provided a positive or negative test result for a given gene, without threshold cycle (*C_T_*) values.

### Detection of CPE.

With *Enterobacterales*, the Revogene Carba C assay detected all KPC, NDM, VIM, IMP, and OXA-48 producers, including OXA-48 variants with carbapenemase activity (OXA-162, -181, -204, and -232), those difficult to detect by phenotypic means (such as OXA-244 and -519) (36, 37), and very recently identified variants (OXA-484, -505, -517, and -793) (Table 1) (38). The chromosomally encoded OXA-535 identified in a *Shewanella* species (39), which has only 91.3% amino acid identity with OXA-48, has not been detected (data not shown). OXA-535, although not present in *Enterobacterales*, was used because it is the progenitor of OXA-436, a distantly related OXA-48 variant responsible for an outbreak associated with several enterobacterial species in Denmark (39, 40). It is thus likely that OXA-436 would not be detected by the Revogene Carba C assay, and further studies on the Revogene Carba C assay should be carried out to test OXA-436 producers. Moreover, OXA-163 and OXA-405, two OXA-48 variants that lack significant imipenemase activity (34, 41) but that show strong expanded-spectrum hydrolytic activity, were consistently not detected by the Revogene Carba C assay. It is still debated whether these enzymes are or are not carbapenemases (35, 42). For example, OXA-163 is considered a carbapenemase by the CLSI, although from an enzymatic point of view with respect to imipenem, these variants are not carbapenemases (43); however, their nucleotide sequences are too similar to that of the *bla*_OXA-48_ gene to be distinguished by most molecular detection assays (24, 28, 30, 33). The Revogene Carba C assay has this particular advantage of distinguishing these *bla*_OXA-48_ variants. Indeed, OXA-163 producers hydrolyze extended-spectrum cephalosporins and are most prevalent in places such as South America, but they need to be individualized as such and not be considered genuine CPE. All the tested IMP variants were correctly identified with Revogene Carba C assay, even those that are not detected by Xpert Carba (Cepheid), such as IMP-2, IMP-8, IMP-11, and IMP-14, or by NG-test Carba5, an immunochromatographic assay, which misses IMP-14 (30, 33, 44). In multiple-carbapenemase producers, all resistance determinants were correctly identified. None of the non-carbapenemase producers or nontargeted-carbapenemase producers yielded positive PCR results.

Using this strain collection (*n* = 118), the overall sensitivity and specificity were 100% (95% confidence interval [95%CI] = 95.0% to 100%) and 100% (95%CI = 83.9% to 100%), respectively, for the detection of CPE (Table 1). Extrapolating these results to the global French CPE epidemiology, the Revogene Carba C assay may be able to detect 99.28% (9,555/9,624) of the CPE identified by the French NRC between 2012 and 2018, missing only 69 CPE (2 GES-5 producers, 10 OXA-23 producers, 2 TMB-1 producers, 1 SME-4 producer, 53 IMI producers, and 1 FRI-1 producer) (Table 4 and data not shown).

**TABLE 4:** Global performance of the Revogene Carba C assay extrapolated on the global French epidemiology of CPOs

Parameter	Result for global French epidemiology		
	CPE (2012–2018)	CP-Pa (2017)	CP-Ab (2017)
Sensitivity, % (95%CI)	99.28 (99.1–99.4)	93.33 (88.4–96.4)	12.40 (951–15.9)
Specificity, % (95%CI)	99.99 (99.8–99.9)	100 (99.1–100)	97.6 (97.6–100)
Expected false positive(s)	1 OXA-405	None	None
Expected false negatives	69 isolates (2 GES-5, 10 OXA-23, 2 TMB-1, 1 SME-4, 53 IMI, 1 FRI)	11 isolates (1 DIM, 10 GES-carbas^*a*^)	378 isolates (OXA-**23**, OXA-**24/40,** and OXA-**58)**
Total no. of carbapenemase producers	9,624 carbapenemase-positive isolates	178 carbapenemase-positive isolates (148 VIM, 3 NDM, 1 KPC, 15 IMP, 10 GES, 1 DIM)	432 carbapenemase-positive isolates (401 OXA-**23**, OXA-**24/40**, and OXA-**58** [92.8%]; 54 NDM [12.5%])

a GES-carbas is a GES β-lactamase with carbapenemase activity.

### Detection of CP-Pa.

Concerning *Pseudomonadaceae*, all KPC, VIM, and NDM producers were perfectly detected (Table 2); however, only 15/17 IMP producers were detected. Two very rare IMP variants, IMP-31 and IMP-46, were not detected. As claimed by the manufacturer, other carbapenemases, such as GES-like carbapenemases (GES-2 and GES-5), GIM-1, AIM-1, SPM-1, DIM-1, and OXA-198, were not detected (Table 2). All non-carbapenemase producers gave negative results. Excluding strains producing these carbapenemases that are not targeted by the assay, the overall sensitivity and specificity were 93.5% (95%CI = 77.1% to 98.9%) and 100% (95%CI = 78.1% to 100%), respectively, for the detection of CP-Pa (Table 2). Of the total collection (*n* = 49) including strains producing an untargeted carbapenemase, the overall sensitivity and specificity were 76.3% (95%CI = 59.4% to 87.9%) and 100% (95%CI = 67.8% to 100%), respectively (Table 2). Extrapolating these results to the global French CP-Pa epidemiology, the Revogene Carba C assay may be able to detect 93.33% (167/178) of the CP-Pa isolates identified by the French NRC in 2017, missing only 11 CP-Pa isolates (10 GES producers and 1 DIM-1 producer) (Table 2) (45).

### Detection of CP-Ab.

Among A. baumannii strains all IMP and NDM producers were perfectly detected (Table 3), resulting in a sensitivity and specificity of 100% (95%CI = 77.0% to 100%) and 100% (95%CI = 82.2% to 100%), respectively, for the targeted carbapenemases in A. baumannii (Table 3). However, as most of the carbapenemases encountered in A. baumannii correspond to nontargeted class D carbapenemases (OXA-23, OXA-40, OXA-58, and OXA-143), when these results are extrapolated to the French epidemiology of CP-Ab, the sensitivity of the assay drops to 12.4% (Table 4) (45).

### Conclusion.

The Revogene Carba C assay showed excellent sensitivity and specificity for the five most common carbapenemases regardless of the host bacteria, including IMP variants that constitute a very heterogeneous family of enzymes and that are not well detected by most molecular or immunochromatographic assays (46). Our study demonstrated that the Revogene Carba C assay is well adapted to the French epidemiology of CPE and CP-Pa, which reflects the epidemiology in many European countries. Its simplicity and short turnaround time make it suitable for use in the routine microbiology laboratory. It can provide results from colonies that grow on Mueller-Hinton (MH) agar but also on those from selective screening media.

The assay detects the main carbapenemases encountered in *Enterobacterales* (KPC, NDM, VIM, IMP, and OXA-48-like) and in P. aeruginosa (VIM, IMP, NDM, and KPC) but only the minor ones in A. baumannii (VIM, IMP, and NDM), as the most widespread OXA carbapenemases usually identified in A. baumannii (OXA-23-like, OXA-24/-40-like, and OXA-58-like) are not targeted by this assay. When extrapolated to the global French epidemiology of CPOs, we would expect (at best) 99.28% sensitivity for CPE detection and 93.33% for CP-Pa but only 12.5% for CP-Ab. Thus, Younden’s index for the French situation would be 99.27, 93.3, and 10 for CPE, CP-Pa, and CP-Ab, respectively.

As for most molecular assays, mutation and/or polymorphisms in the primer/probe binding region of the targeted gene may lead to false-negative results, and thus some variants may not be detected. It is of outmost importance that a molecular test be evaluated on the local epidemiology in order to know which are the alleles that might be missed. Regular evaluation of novel variants is required to assess the sensitivity and specificity of this assay in a constantly evolving carbapenemase field. The main drawback of this assay is that it so far has been validated only on pure cultures. Evaluation directly on rectal swabs is a mandatory next step for the assay to be fully useful for carbapenemase gene detection.
